# Elevated 17β-Estradiol Protects Females from Influenza A Virus Pathogenesis by Suppressing Inflammatory Responses

**DOI:** 10.1371/journal.ppat.1002149

**Published:** 2011-07-28

**Authors:** Dionne P. Robinson, Maria E. Lorenzo, William Jian, Sabra L. Klein

**Affiliations:** W. Harry Feinstone Department of Molecular Microbiology and Immunology, The Johns Hopkins Bloomberg School of Public Health, Baltimore, Maryland, United States of America; National Institutes of Health, United States of America

## Abstract

Studies of the 1918 H1N1 influenza pandemic, the H5N1 avian influenza outbreak, and the 2009 H1N1 pandemic illustrate that sex and pregnancy contribute to severe outcome from infection, suggesting a role for sex steroids. To test the hypothesis that the sexes respond differently to influenza, the pathogenesis of influenza A virus infection was investigated in adult male and female C57BL/6 mice. Influenza infection reduced reproductive function in females and resulted in greater body mass loss, hypothermia, and mortality in females than males. Whereas lung virus titers were similar between the sexes, females had higher induction of proinflammatory cytokines and chemokines, including TNF-α, IFN-γ, IL-6, and CCL2, in their lungs than males. Removal of the gonads in both sexes eliminated the sex difference in influenza pathogenesis. Manipulation of testosterone or dihydrotestosterone concentrations in males did not significantly impact virus pathogenesis. Conversely, females administered high doses of estradiol had a ≥10-fold lower induction of TNF-α and CCL2 in the lungs and increased rates of survival as compared with females that had either low or no estradiol. The protective effects of estradiol on proinflammatory cytokines and chemokines, morbidity, and mortality were primarily mediated by signaling through estrogen receptor α (ERα). In summary, females suffer a worse outcome from influenza A virus infection than males, which can be reversed by administration of high doses of estradiol to females and reflects differences in the induction of proinflammatory responses and not in virus load.

## Author Summary

Sex and pregnancy affect the outcome of infection with seasonal, avian, and pandemic influenza viruses among young adults. Males and females are biologically different, yet the implications of these differences on influenza A virus pathogenesis are not well characterized. Generally, females mount more robust immune responses to viral challenge than males, which can result in more efficient virus clearance at the cost of developing immune-mediated pathology. In this study, we tested the hypothesis that sex and sex steroid hormones differentially impact the outcome of influenza A virus infection in mice. Our data illustrate that influenza A virus dysregulates reproductive function as well as cytokine and chemokine production in females, rendering them significantly more susceptible to weight loss, hypothermia, and death than males. Administration of a high dose of estradiol or an estrogen receptor α agonist to females suppresses the excessive induction of cytokines and chemokines and increases survival following infection. The protective effects of estradiol on influenza pathogenesis reflect changes in the induction of proinflammatory responses and not in virus load. Uncovering the mechanisms mediating how sex and sex steroid hormones affect influenza pathogenesis may result in preventative measures and treatments that are optimized for both sexes.

## Introduction

Males and females differ in their responses to infection with many viral pathogens, including human immunodeficiency virus (HIV), herpes simplex viruses, and hantaviruses [1]. Although societal and behavioral factors can influence exposure to viruses and access to vaccines and treatments for infection [2], genetic and physiological differences between the sexes can cause differential immune responses to viruses [3]. Because females tend to mount higher innate [4], [5], cell-mediated [5], [6], [7], and humoral [8] immune responses than males, viral loads are often reduced among females [1]. Heightened immunity in females also can lead to the development of immunopathology following viral infection [5]. Elevated immunity in females represents a balance between immune responses conferring protection and causing pathology.

Growing evidence links sex differences in immune function with circulating sex steroid hormones [9], [10]. Receptors for sex steroids are expressed in a variety of lymphoid cells [11], [12]. Androgens, including dihydrotestosterone (DHT) and testosterone (T), suppress the activity of immune cells [9], [13], [14]. Estradiol (E2) can have divergent effects, with low doses enhancing proinflammatory cytokine production (e.g., IL-1, IL-6, and TNF-α) and T helper cell type 1 (Th1) responses and high or sustained concentrations reducing production of proinflammatory cytokines and augmenting Th2 responses and humoral immunity [15]. Elevated E2 also attenuates production of CXC chemokine ligand (CXCL)-8, CXCL10, chemokine (C-C motif) ligand 2 (CCL2), and CCL20 and recruitment of leukocytes and monocytes into several tissues, including the lungs [16], [17], [18], [19]. The anti-inflammatory effects of high E2 are mediated by signaling through estrogen receptors (ERs), which inhibits activation of NF-κB-mediated inflammatory responses [20].

Observational data for influenza reveal that the outcome of pandemic influenza as well as avian H5N1 is generally worse for young adult females [21]. In the United States, during the 1957 H2N2 pandemic, mortality was higher among females than males 1–44 years of age [22]. Worldwide as of 2008, females were 1.6 times less likely to survive H5N1 infection than males [23]. During the 2009 H1N1 pandemic, a significant majority of patients hospitalized with severe 2009 H1N1 disease were young adult females (15–49 years of age) [21], [24], [25], [26], [27], [28]. Pregnancy and other risk factors (e.g., asthma and chronic obstructive pulmonary disorder) contribute to the severity of disease in females [21].

The mechanisms mediating how the sexes differ in response to influenza virus infection as well as the effects of sex steroids on influenza pathogenesis remain largely undefined. We hypothesize that biological differences in immune responses may explain variation between the sexes during influenza virus infection. Several studies reveal that excessive proinflammatory responses (i.e., the cytokine storm) contribute significantly to morbidity and mortality from influenza virus infection [29], [30], [31], [32], [33]. Our data reveal that females experience greater morbidity and mortality than males, which can be reversed by administration of exogenous E2 or an ERα agonist to females. Our data further indicate that sex differences and the effects of E2 on influenza pathogenesis reflect differences in the production of proinflammatory cytokines and chemokines as opposed to differences in virus load.

## Results

### Morbidity and mortality from H1N1 influenza is greater in females than males

**Figure 1:**
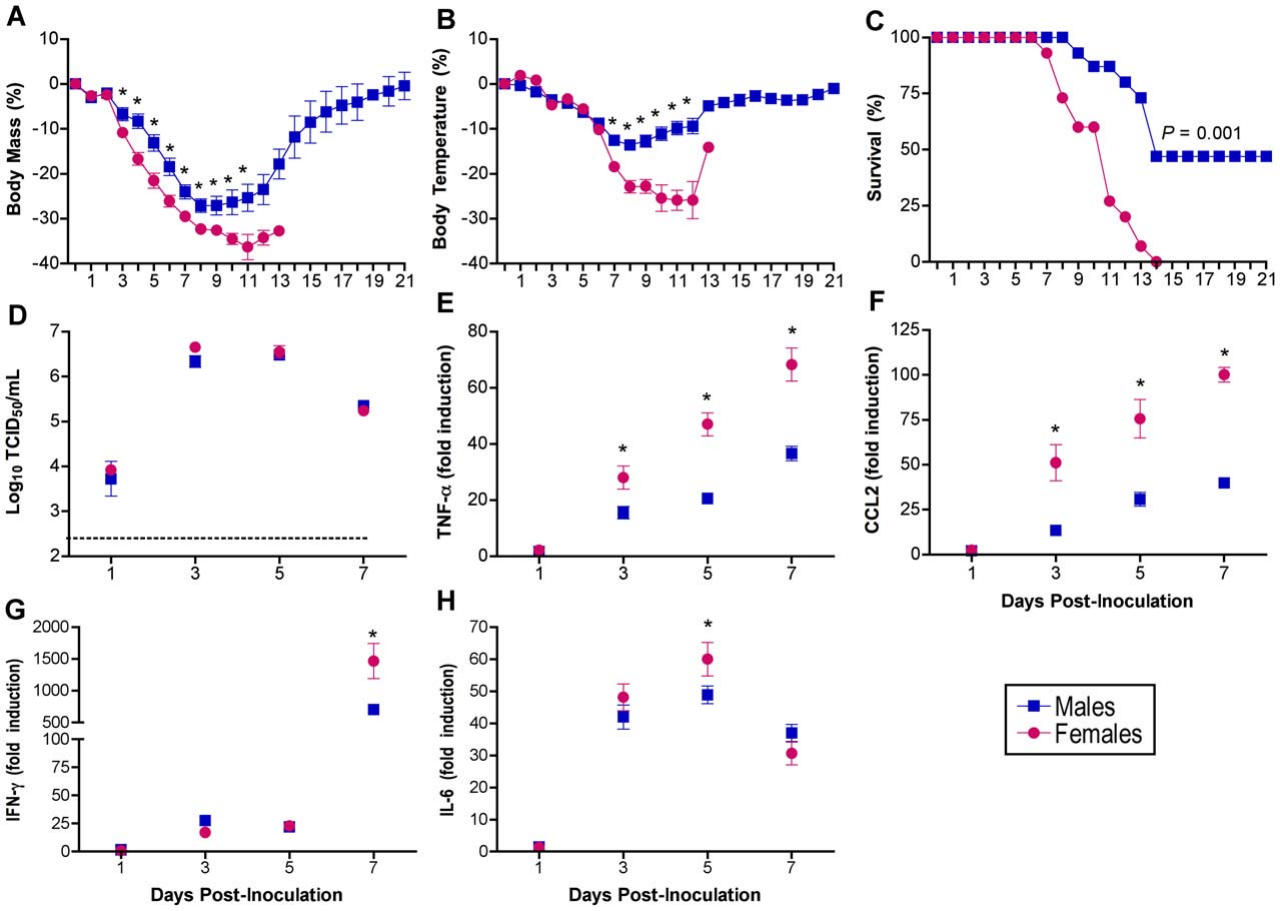
Influenza virus infection causes greater morbidity and mortality in females than males. Male and female mice were inoculated with PR8 and monitored for changes in body mass (A), rectal temperature (B), and survival (C) for up to 21 days (n = 15/sex). Infectious virus titers (D) as well as concentrations of TNF-α (E), CCL2 (F), IFN-γ (G), and IL-6 (H) were measured in homogenates of lungs removed 0, 1, 3, 5, or 7 days p.i. (n = 10–15/sex/time-point). Fold change represents concentrations of proteins at Days 1–7 p.i. relative to concentrations at Day 0. Data represent the mean ± SEM. The dotted line in panel D represents the limit of detection for the assay. Significant differences between the sexes as determined by post hoc analyses at each time-point are represented by an asterisk (*), *P*<0.05.

To examine whether the sexes respond differently to influenza A virus infection, adult male and female C57BL/6 mice were inoculated with 10^2^ TCID_50_ of influenza A/Puerto Rico/8/1934 (PR8; H1N1) and monitored daily for changes in morbidity and mortality for 21 days. Females showed a greater percent reduction in body mass (Fig. 1A) and body temperature (Fig. 1B) than males, with these differences being most pronounced 7–13 days post-inoculation (p.i.) (MANOVA sex x day *P*<0.0001 in each case). Survival following influenza infection was significantly reduced in females compared with males (log rank *P*<0.001), in which no females survived infection with PR8, whereas 47% of the males survived through 21 days p.i. (Fig. 1C; Χ^2^ *P*<0.05). The average day of death was 2 days earlier for females (9.4±0.6 days) than males (11.5±0.7 days) (t-test *P*<0.05).

Titers of infectious virus peaked 3–5 days after infection, but sex differences were not observed (Fig. 1D), suggesting that changes in virus load alone were not responsible for the observed sex differences in morbidity and mortality. Highly pathogenic influenza viruses cause severe disease by initiating profound proinflammatory cytokine and chemokine responses [29], [33]. Inflammatory cytokine responses increased in both sexes, in a time-dependent manner as documented previously [30], [34], [35], [36]. Interleukin-1β, IL-12p70, IL-10, and TGF-β were induced within 24 h p.i.; IFN-β, IL-6, TNF-α, CCL2, and CCL3 were induced within 72 h p.i.; and IFN-γ and IL-10 were induced 7 days p.i. in both sexes (Table S1; 2-way ANOVAs, main effect of day *P*<0.05). Females showed a greater induction of CCL2, TNF-α, IFN-γ, and IL-6 than males (Fig. 1E-H, 2-way ANOVAs sex x day *P*<0.05).

### Influenza virus infection suppresses reproductive function in both sexes

**Figure 2:**
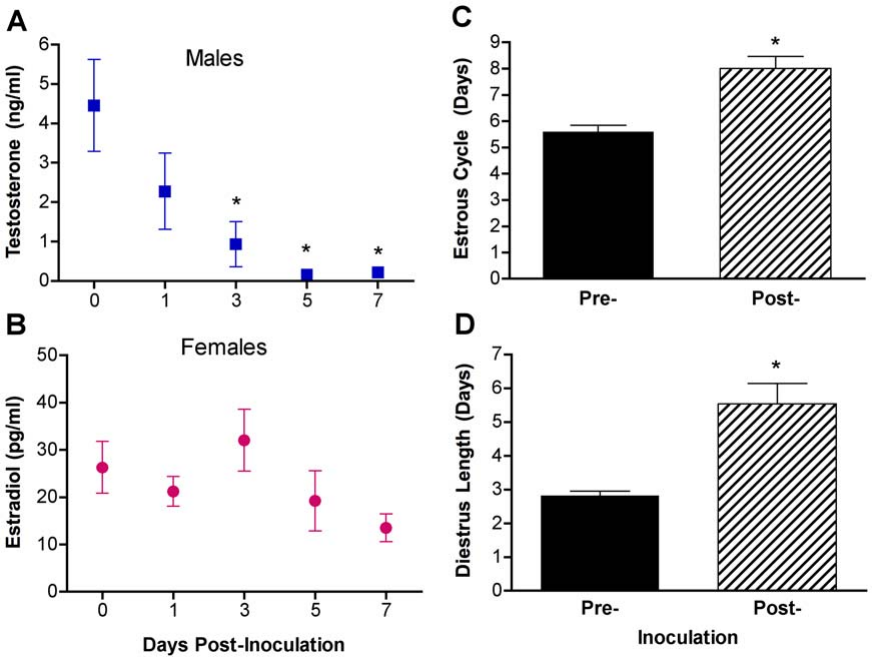
Influenza virus infection alters sex steroid concentrations and reproductive function in males and females. Male and female mice (n = 10/sex/time-point) were inoculated with PR8, serum was collected, and T concentrations in males (A) and E2 concentrations in females (B) were analyzed Days 0, 1, 3, 5, or 7 p.i. To assess estrous cycles, females (n = 16) were sampled daily by vaginal lavage before and after inoculation with PR8 and the duration of the estrous cycle (C) and diestrus (D) was quantified. For sex steroid hormone measurements, plasma from uninfected animals were collected at the same time as other samples and designated as Day 0 p.i. Data represent the mean ± SEM. Significant differences between Day 0 and other time-points p.i. based on post hoc analyses are represented by an asterisk (*), *P*<0.05.

Inflammatory immune responses induced by fatal infection with pathogens (e.g., HIV) affect the brain to reduce reproductive function, appetite, and thermoregulation [37], [38], which in mice can result in greater suppression of reproductive activity in females than males [39]. To evaluate the effects of PR8 infection on reproductive physiology, T concentrations in males and E2 concentrations in females were evaluated in plasma samples collected from separate mice at several time-points during the first week p.i. Infection of males reduced circulating T concentrations on days 3–7 p.i. as compared with uninfected males (Fig. 2A, 1-way ANOVA *P*<0.05).

In females, infection with PR8 appeared to cause persistently low E2 concentrations (Fig. 2B); single time-point sampling of cyclical hormones, however, is difficult to accurately evaluate in females [40]. To better characterize the hormonal milieu of females during influenza virus infection, we monitored the estrous cycles of female mice before and after infection with PR8. The average duration of the estrous cycle increased significantly following influenza virus infection (Fig. 2C; paired t-test *P*<0.05) and this increase in estrous cycle length was attributed to an increase in the duration of diestrus (Fig. 2D; paired t-test *P*<0.05). As diestrus is the stage of the estrus cycle that corresponds with the follicular phase, when both estrogens and progesterone are at their nadir [40], these data suggest that influenza virus infection suppresses ovarian function in females and results in persistently low circulating E2.

### Removal of the gonads reduces sex differences in influenza pathogenesis

**Figure 3:**
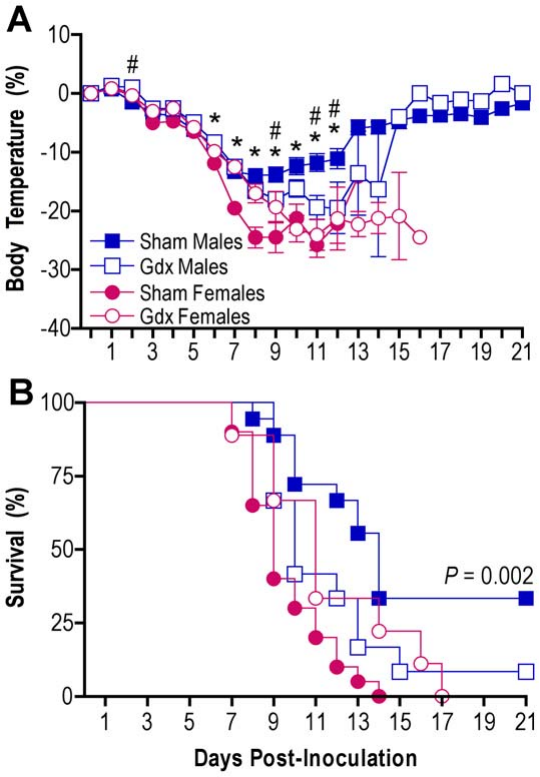
Removal of the gonads reduces the sex difference in influenza pathogenesis. Rectal temperature (A) and survival (B) were monitored for 21 days after inoculation of intact (sham) male (n = 18), sham female (n = 20), gonadectomized (gdx) male (n = 12), or gdx female (n = 9) mice with PR8. Data represent the mean ± SEM. Significant differences between sham males and females as determined by post hoc analyses at each time-point are represented by an asterisk (*), *P*<0.05. Significant differences between intact and gdx males as determined by post hoc analyses at each time-point are represented by a number symbol (#), *P*<0.05.

To establish whether gonadal secretions modulate sex differences in influenza pathogenesis, we compared morbidity and mortality following PR8 infection in gonadally-intact (sham) and gonadectomized (gdx) male and female mice. Consistent with previous data (Fig. 1), hypothermia (MANOVA sex x day *P*<0.0001) and mortality (log rank *P*<0.001) following influenza virus infection were greater among gonadally-intact (sham) females than gonadally-intact (sham) males (Fig. 3A and B). Gonadectomy of males resulted in more pronounced hypothermia (MANOVA treatment x day *P*<0.003) and death (log rank *P* = 0.04) as compared with gonadally-intact males (Fig. 3A and B). Mortality (log rank *P* = 0.046), but not morbidity, was lower among gdx than gonadally-intact females during influenza virus infection. Among gdx animals, removal of the gonads in male and female mice eliminated the dimorphism in hypothermia and survival (Fig. 3A and B). In summary, sex differences in influenza pathogenesis are partially mediated by the presence of gonadal secretions.

### Estradiol treatment protects females against lethal influenza infection by suppressing proinflammatory responses

**Figure 4:**
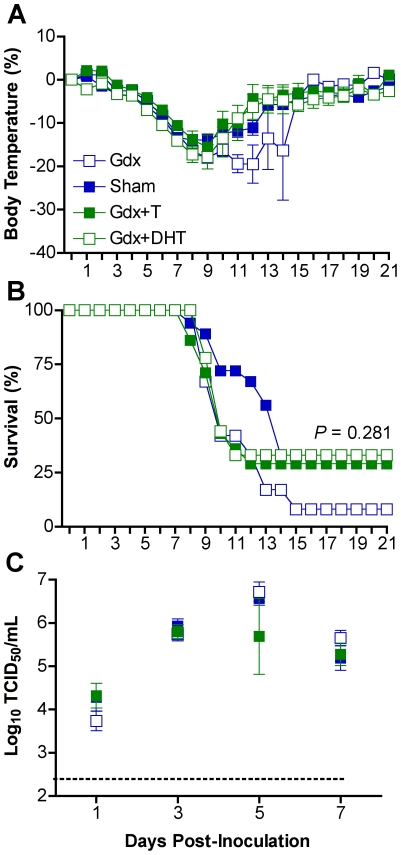
Androgen replacement does not significantly affect morbidity or mortality from influenza virus infection in males. Males were left intact (sham; n = 10–18/time-point/experiment) or gonadectomized (gdx) and implanted with silastic capsules that were empty (n = 9–12/time-point/experiment) or filled with testosterone (T; n = 8–14/time-point/experiment) or dihydrotestosterone (DHT; n = 10). Males were inoculated with PR8 and monitored daily for changes in body temperature (A) and survival (B). Infectious virus titers (C) were measured by TCID_50_ in lungs removed Days 1, 3, 5, or 7 p.i. The dotted line in panel C represents the limit of detection for the assay. Data represent the mean ± SEM.

To establish whether androgens in males and estrogens in females affect responses to influenza, we examined the effects of removal and replacement of sex steroids on influenza pathogenesis. Among males, gdx animals that received exogenous T had greater concentrations of circulating T (14.74±0.65 ng/ml) than gdx males (1.00±0.01 ng/ml) (t-test *P*<0.05). Treatment with either T or DHT did not significantly reverse the effects of gdx on either hypothermia (Fig. 4A) or mortality (Fig. 4C). Titers of PR8 also did not differ among hormonally-manipulated and gonadally-intact males (Fig. 4D). Manipulation of androgens in males affected concentrations of CCL3, IFN-γ, and IL-10, but not in a discernable pattern associated with morbidity and mortality (Table S2; 2-way ANOVAs treatment x day *P*<0.05).

**Figure 5:**
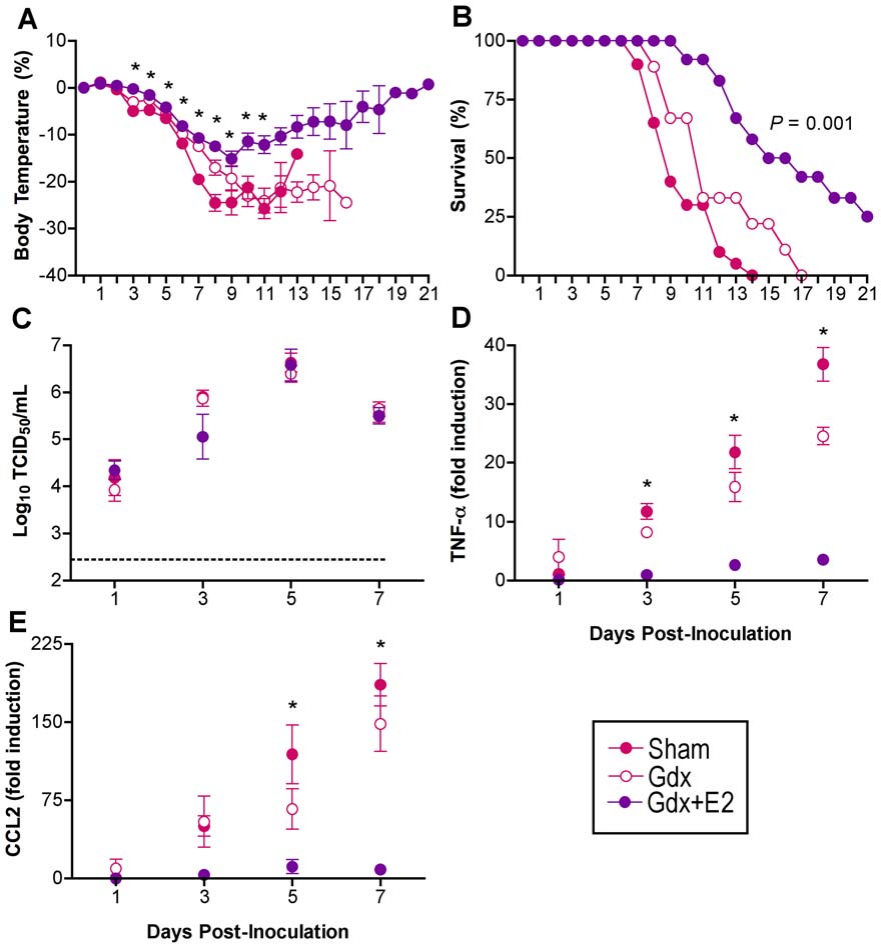
Exogenous E2 protects females against influenza virus infection. Females were intact (sham; n = 9–20/time-point/experiment), gonadectomized (gdx; n = 7–10/time-point/experiment), or gdx and treated with E2 (n = 8–12/time-point/experiment). Females were inoculated with PR8 and monitored daily for changes in rectal temperature (A) and survival (B). Infectious virus titers (C) as well as concentrations of TNF-α (D) and CCL2 (E) were measured in lungs removed Days 1, 3, 5, or 7 p.i. Fold change represents concentrations of proteins at Days 1–7 p.i. relative to concentrations at Day 0. Data represent the mean ± SEM. The dotted line in panel C represents the limit of detection for the assay. Significant differences between E2-treated and sham or gdx females, as determined by post hoc analyses, are represented by an asterisk (*), *P*<0.05.

Among females, those that received exogenous E2 had significantly higher serum concentrations of E2 (978±39 pg/ml) than gdx females (1±26 pg/ml) (t-test *P*<0.05). Administration of exogenous E2 mitigated hypothermia (Fig. 5A; MANOVA treatment x day *P*<0.005) and mortality (Fig. 5B; log rank *P*<0.001) following PR8 infection as compared with gonadally-intact and gdx female mice. Females that received E2 were more likely to survive PR8 infection and those that died had a later day of death (12.8±1.2 days) than did sham (8.6±0.4 days) or gdx (10.7±1.1 days) female mice (1-way ANOVA *P*<0.05). Administration of E2 did not affect virus replication kinetics (Fig. 5C), but diminished the rise in TNF-α and CCL2 in the lungs that was apparent among gonadally-intact and gdx female mice (Fig. 5D and E; 2-way ANOVA treatment x sex *P*<0.001). Although hormone manipulation in females altered other cytokines, including IFN-γ, IL-10, IL-12(p70), and CCL3 (Table S3; 2-way ANOVAs treatment x sex *P*<0.05), the patterns were not associated with changes in morbidity or mortality. In summary, females with low (sham) or no (gdx) circulating E2 suffer a worse outcome from infection and have higher proinflammatory responses than females with high E2.

### Estradiol protects against lethal influenza by signaling through ERα

**Figure 6:**
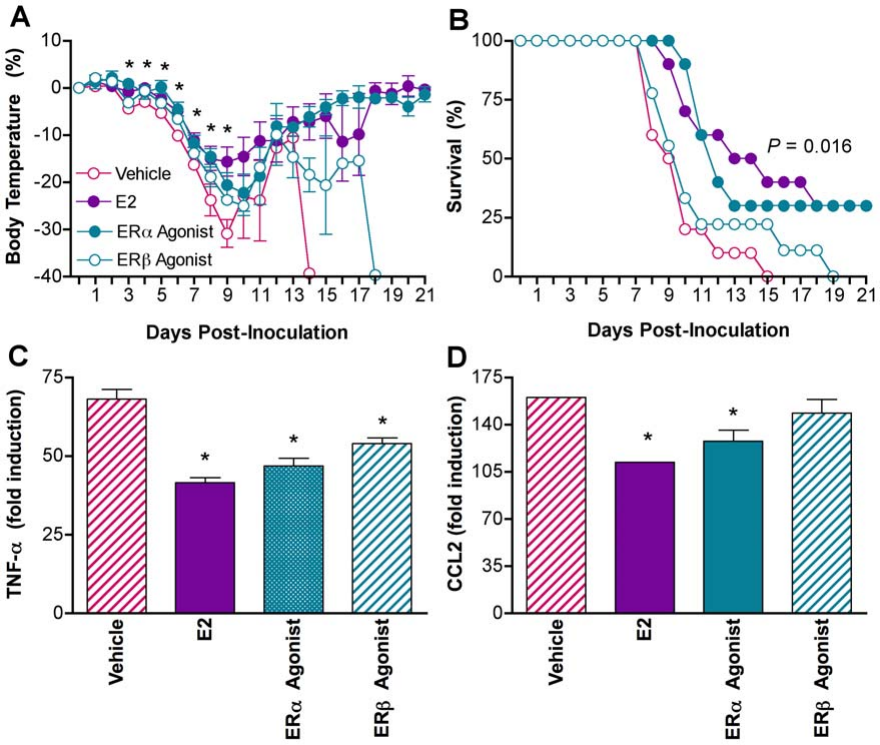
Estradiol protects females against influenza through ERα signaling. Females were gonadectomized (gdx) and assigned to received vehicle (n = 9–12/time-point/experiment), E2 (n = 9–10/time-point/experiment), the ERα agonist (PPT; n = 9–10/time-point/treatment), or the ERβ agonist (DPN; n = 8–9/time-point/experiment). Females were inoculated with PR8 and monitored daily for changes in rectal temperature (A) and survival (B). Concentrations of TNF-α (C) and CCL2 (D) were quantified in lung homogenates collected from females at Days 0 and 7 p.i. Fold change represents concentrations of proteins at Day 7 p.i. relative to concentrations at Day 0. Data represent the mean ± SEM. Significant differences between treatment groups and vehicle controls, as determined by post hoc analyses, are represented by an asterisk (*), *P*<0.05.

The anti-inflammatory effects of high E2 are mediated by signaling through two nuclear receptors, ERα and ERβ [41], which antagonizes nuclear factor kappa B (NF-κB) activity [20]. To determine which ER was mediating the effects of E2 on influenza pathogenesis, gdx females were administered E2, vehicle, or vehicle containing agonists specific to ERα (Propylpyrazole-triol; PPT) or ERβ (diarylpropionitrile; DPN). Treatment with the ERα agonist, but not vehicle or the ERβ agonist, reduced hypothermia (Fig. 6A; MANOVA treatment x day *P*<0.0001) and increased rates of survival (Fig. 6B; log rank *P*<0.01) to levels that were similar to females treated with E2. Titers of PR8 in the lungs peaked for all females at Day 3 p.i., but were not affected by ER manipulation (data not shown). Treatment with the ERα agonist reduced TNF-α and CCL2 (Fig. 6C and D; 1-way ANOVA *P*<0.001) in the lungs to levels that were similar to those of females treated with E2. Treatment with the ERβ agonist reduced TNF-α (Fig. 6C; 1-way ANOVA *P*<0.001), but not CCL2 (Fig. 6D) as compared with vehicle-treated females. Although administration of the ERα and ERβ agonists altered IL-6 and IL-10, these patterns were not correlated with morbidity and mortality (Table S4; 1-way ANOVAs *P*<0.05). The effects of E2 on proinflammatory responses to infection, in particular CCL2 responses, and disease outcome are primarily mediated by signaling through ERα.

## Discussion

Although epidemiological data suggest that females experience more severe disease and suffer a worse outcome from influenza virus infection than males [21], whether these differences reflect sex or gender is difficult to assess as both factors can affect exposure and vulnerability to influenza A viruses [21]. Using a small animal model, our data and data from others [42] illustrate that there are distinct biological differences in how males and females respond to influenza.

Disease associated with highly pathogenic influenza viruses and the clinical manifestations that ensue in humans can be mediated by the proinflammatory response (e.g., TNF-α, IL-6, CCL2, CCL3, and CXCL10) initiated by the host in response to infection [29], [30], [31], [32], [33]. Studies of patients infected with avian influenza viruses further reveal that higher proinflammatory responses are correlated with mortality during infection [33]. Elevated production of CCL3 and CCL2 and expression of CCR2 recruit monocytes and neutrophils into the lungs and regulate inflammation and influenza A virus replication [43]. The data from the present study illustrate that inflammatory immune responses, including induction of CCL2, IFN-γ, IL-6, and TNF-α, are elevated in the lungs of females compared with males. Infectious virus titers, however, do not differ between the sexes and are not altered by hormones. Similarly, infection of adult BALB/c mice with a mouse-adapted H3N1 influenza A virus results in greater lung hyperresponsiveness to methacholine challenge and production of CCL2, but not virus titers, in females compared with males [42]. These data support and extend the hypothesis that host-mediated immunopathology rather than virus replication underlies influenza pathogenesis.

Sex differences in disease outcome are likely mediated by multiple factors, including sex steroids, glucocorticoids, and the direct activity of sex chromosomal genes [44]. In the present study, removal of gonadal secretions in both males and females reduced the sex difference in morbidity and mortality, illustrating that the sex difference in influenza pathogenesis is reversible and that activational sex steroids in adulthood affect the outcome of infection. Furthermore, sex differences in response to influenza A virus infection are not observed among pre-pubertal mice [42]. Within males, however, manipulation of androgens did not significantly affect influenza pathogenesis, suggesting that some androgenic effects may be organized early during sexual differentiation. The extent to which sex differences in immunity are hard-wired early during development must be considered [44].

Our data reveal that estrogens are one mechanism mediating influenza pathogenesis in females. Infection with influenza virus disrupted reproductive function in gonadally-intact females, resulting in a prolonged state of diestrus, which is the stage of the reproductive cycle when E2 and progesterone concentrations are at their lowest [40]. Gonadectomized females (i.e., females with no circulating E2) and gonadally-intact females (i.e., females with low circulating E2 as a result of infection) produced higher inflammatory responses and suffered a worse outcome from infection than gdx females administered exogenous E2. These data support the hypothesis that low concentrations of E2 in females promote excessive inflammatory responses that contribute to disease pathogenesis [15].

In the present study, exogenous administration of E2 reduced the induction of pulmonary inflammatory responses and protected females against influenza. High doses of estrogens also are protective in animal models of multiple sclerosis (MS), in which supraphysiological doses of estrogens reduce inflammatory responses and progression of this autoimmune disease [45], [46]. In contrast, low cyclical levels of estrogens in gonadally-intact females have little effect on the outcome of MS. High E2 has potent anti-inflammatory actions, including repression of proinflammatory gene transcription and cytokine production [11], [47], which is partially mediated by inhibition of NF-κB transcriptional activity [48]. The anti-inflammatory effects of estrogens have been observed in several models for diseases, including autoimmunity, atherosclerosis, arthritis, inflammatory bowel disease, and asthma [15]; our data reveal that influenza is another disease of public health importance that is influenced by estrogens.

The proinflammatory effects of low or no E2 and the anti-inflammatory effects of high E2 in females are mediated by signaling through the ER, which regulates the activity of NF-κB [20]. Administration of an ERα, but not an ERβ, agonist protected females against influenza infection. ERα has been identified in several immune cells, including DCs, macrophages, and T cells, whereas ERβ is expressed in epithelial cells, macrophages, and B cells [11], [41]. The differential effects of ERα and ERβ agonists in vivo provide insight into the cell types that may be responsible for the exacerbated inflammatory responses observed in influenza-infected females with low or no E2.

Our data suggest a number of important avenues of research that require further investigation. Mechanisms in addition to low circulating E2 likely influence sex differences in influenza pathogenesis and we are actively investigating the effect of other sex-specific factors on viral disease. In addition to the mouse-adapted PR8 (H1N1), sex differences are reported in response to mouse-adapted H3N1 [42] and H3N2 (Lorenzo et al. unpublished data); mouse models utilizing mouse-adapted influenza A viruses, however, may not completely reflect virus pathogenesis in humans. Because clinical isolates of influenza A viruses cause limited pathology in mice [49], [50], [51], examination of sex differences and the effects of sex steroids in response to non-adapted strains of influenza in mice would need to be limited to highly pathogenic viruses such as the 1918 virus strain and avian H5N1 viruses. Alternatively, sex differences in response to infection with clinical isolates of influenza A viruses could be evaluated in alternative animal models, such as ferrets [49]. The use of mouse-adapted strains of influenza to demonstrate sex differences and effects of sex steroids on influenza pathogenesis in mice reveal significant differences and suggest that these differences should be considered in evaluations of epidemiological and clinical human data. The observation that elevated E2 reduces, rather than elevates, the severity of influenza A virus infection does not explain why pregnancy is associated with worse outcome after infection. Elevation of E2 concentrations in non-pregnant females does not completely recapitulate pregnancy as several other hormones, including progesterone, estriol, and glucocorticoids also dramatically change during pregnancy and can impact immune function [52], [53]. While sex steroid-modulation of influenza pathogenesis likely contributes to the increased severity of disease during pregnancy, the data from the current study suggest that E2 is not the mechanism mediating severe outcome of infection during pregnancy.

There is clinical relevance to uncovering the mechanisms mediating how sex and sex steroids affect responses to influenza viruses as this may result in preventative measures and treatments that are optimized for each sex. Most epidemiological and clinical studies of influenza in humans do not partition or analyze data by sex and a majority of animal studies of influenza either use only females or do not report the sex of their animals [21]. The data from the present study provide evidence that the pathogenesis of influenza virus infection differs between the sexes and is influenced by the effects of sex hormones on inflammatory immune responses.

## Materials and Methods

### Animals

Adult (6–8 weeks old) male (total n = 308) and female (total n = 356) C57BL/6 mice were purchased from NCI Frederick, housed 5/microisolater cage with food and water available *ad libitum*, and handled using Biosafety Level (BSL)-2 practices.

### Ethics statement

All experiments were performed in compliance with the standards outlined in the National Research Council's *Guide to the Care and Use of Laboratory Animals*. The animal protocol (MO09H26) was reviewed and approved by the Johns Hopkins University Animal Care and Use Committee. All efforts were made to minimize animal suffering.

### Surgical procedures

Male and female mice were anesthetized with 2.5% isoflurane (Baxter Healthcare Corporation, Deerfield, IL) mixed with oxygen and bilaterally gonadectomized as previously described [54], [55], [56]. All animals were given two weeks to recover prior to infection.

### Vaginal cell cytology

Vaginal cell samples were collected at 1600–1700 h, smeared onto clean glass slides, fixed, stained with Diff-Quick Staining kit (Andwin Scientific, Addison, IL), and diagnosed for stage of estrus based on the cellular profile of each sample: proestrus (80–100% intact, healthy epithelial cells), estrus (100% cornified epithelial cells), diestrus I (∼50% cornified epithelial cells and 50% leukocytes), and diestrus II (80–100% leukocytes) [57], [58]. Only females that exhibited at least 3 regular estrous cycles (16/20) prior to infection were included.

### Sex hormone capsules

Hormone and placebo capsules were made with 10 mm of silastic tubing (inner diameter  = 0.04 in; outer diameter  = 0.085 in; VWR International, Bridgeport, NJ) and sealed with silastic medical adhesive (Dow Corning, Midland, MI). Hormone capsules were left empty (placebo) or filled with 5 mm of testosterone (T), dihydrotestosterone (DHT), or 17β-estradiol (E2) purchased from Sigma Aldrich (St. Louis, MO) and sealed with 2.5 mm of adhesive at either end [56]. Capsules were incubated in 0.9% saline at 37°C overnight prior to implantation.

### Estrogen receptor agonists

Propylpyrazole-triol (PPT) and diarylpropionitrile (DPN) were purchased from Tocris Bioscience (Ellisville, MO), suspended in Miglyol 812N oil (kindly provided by Sasol, Hamburg, Germany), and administered at 10 mg/kg and 8 mg/kg, respectively [59]. Animals received daily subcutaneous injections of either vehicle (90% Miglyol, 10% EtOH) or vehicle containing PPT or DPN.

### Sample collection

For experiments in which morbidity and mortality were monitored for up to 21 days post-inoculation, animals were not euthanized as death was an approved endpoint in our IACUC protocol. Body mass and rectal temperature were measured daily between 0800 and 1000 h. Animals were weighed to the nearest hundredth of a gram and rectal temperature was monitored with a Thermalert TH-5 monitor (25°C–45°C) and RET-3 rectal microprobe for mice (Physitemp Instruments, Inc., NJ), which stably evaluates body temperature to the nearest 0.1°C in 3–5 seconds. For time course experiments, animals were randomly assigned to be euthanized at 0, 1, 3, 5, or 7 days p.i., at which time they were anesthetized with isoflurane and terminally bled from the retro-orbital sinus into heparinized tubes and plasma was stored at −80°C and used to measure hormones. Whole lungs were collected, snap-frozen, and stored at −80°C until homogenized in Dulbecco's Modified Eagles Medium (DMEM) supplemented with 1% Penicillin/Streptomycin and 1% L-glutamine (Invitrogen, Carlsbad, CA). Homogenates were centrifuged and the supernatants were stored at −80°C and used to measure virus titers and cytokine concentrations.

### Virus infection and quantification

Mouse-adapted influenza A virus, A/Puerto Rico/8/34 (PR8) was provided by Dr. Maryna Eichelberger at the Food and Drug Administration. Mice were anesthetized with Ketamine/Xylazine (80 mg/kg and 6 mg/kg, respectively) and intranasally inoculated with 30 µl of vehicle (DMEM) or 102 50% tissue culture infective dose (TCID_50_) units of PR8 diluted in DMEM (which corresponds to 1.24 50% mouse lethal dose (MLD_50_) for males and 0.78 MLD50 for females, based on the Reed and Meunch method) between 0800 and 1000 h. The challenge dose was selected based on previous experiments that quantified the lethal dose that killed 50% of the animals (LD_50_). Virus quantification was performed using the TCID_50_ method measuring cytopathic effects (CPE) of influenza A virus on a monolayer of Madin Darby Canine Kidney (MDCK) cells [60].

### Sex hormone auantification

Hormones were concentrated from plasma by ether extraction and hormone quantification was performed using testosterone and estradiol enzyme immunosorbant assays (EIA) purchased from Cayman Chemicals (Ann Arbor, MI).

### Cytokine quantification

Supernatants from lung homogenates were used to measure CCL3, IFN-β, IL-1β, and TGF-β by ELISAs (R&D Systems, BD Biosciences, PBL Biomedical Laboratories) and CCL2, IL-12(p70), TNF-α, INF-γ, IL-10, and IL-6 with the mouse inflammation cytometric bead array (BD Biosciences).

### Statistical analysis

Kaplan Meier survival curves were compared using log rank analyses. The proportion of animals that survived influenza A virus infection was compared among experimental groups using χ^2^ analyses. Morbidity data were analyzed with multivariate ANOVAs (MANOVAs) with one within-subjects variable (days) and one between-subjects variable (sex or treatment) and significant interactions were further analyzed using planned comparisons. Virus titers and protein concentrations were analyzed with 2-way ANOVAs with day p.i. and sex/treatment as the independent variables and significant interactions were further analyzed using the Bonferroni method for pairwise multiple comparisons. Hormone concentrations were analyzed by t-tests or 1-way ANOVAs followed by Bonferroni post hoc tests. Changes in estrous cycle length were evaluated using paired t-tests. Mean differences were considered statistically significant if *P*<0.05 (SYSTAT 13, Systat Software, Chicago, IL).

## Supporting Information

Table S1 Fold induction of cytokines and chemokines in the lungs of gonadally intact male and female mice.
(DOC)

Table S2 Fold induction of cytokines and chemokines in the lungs of males that were gonadally intact, gonadectomized, or gonadectomized with testosterone replaced.
(DOC)

Table S3 Fold induction of cytokines and chemokines in the lungs of females that were gonadally intact, gonadectomized, or gonadectomized with estradiol replaced.
(DOC)

Table S4 Fold induction of cytokines and chemokines in the lungs of females that were gonadectomized and assigned to receive vehicle, estradiol, an ERα agonist, or an ERβ agonist.
(DOC)
