# Supplementary material for: Elevated 17β-Estradiol Protects Females from Influenza A Virus Pathogenesis by Suppressing Inflammatory Responses
Source: PLoS Pathog. 2011 Jul 28;7(7):e1002149. doi: 10.1371/journal.ppat.1002149 (PMC3145801; doi:10.1371/journal.ppat.1002149)
Supplement: Table S1 — Fold induction of cytokines and chemokines in the lungs of gonadally intact male and female mice. (DOC) [file ppat.1002149.s001.doc]

**Supporting Information**

**Table S1. Fold induction of cytokines and chemokines in lung homogenates from gonadally intact males and females**.

|  |  | **Days Post-Inoculation** | | | |
| --- | --- | --- | --- | --- | --- |
| **Cytokine** | **Sex** | 1 | 3 | 5 | 7 |
| CCL2 | Males | 2.01±0.37 ^a^ | 13.46±1.63 ^a^ | 30.74±3.86 ^b^ | 39.83±1.13 ^b^ |
|  | Females | 2.42±0.33 ^a^ | 51.11±10.04* ^b^ | 75.61±10.77* ^b^ | 100.11±4.10* ^c^ |
| CCL3 | Males | 1.53±0.25 ^a^ | 9.16±1.39 ^a^ | 24.96±5.54 ^b^ | 80.37±9.66 ^c^ |
|  | Females | 0.77±0.12 ^a^ | 4.80±0.44 ^a^ | 15.67±3.36 ^a^ | 66.59±7.46 ^b^ |
| IFN-β | Males | 1.02±0.31 ^a^ | 6.86±1.03 ^b^ | 7.83±0.82 ^b^ | 5.61±0.47 ^b^ |
|  | Females | 1.47±0.17 ^a^ | 6.00±0.50 ^a^ | 9.12±0.82 ^c^ | 4.66±0.60 ^b^ |
| IFN-γ | Males | 1.50±0.41 ^a^ | 27.57±4.67 ^a^ | 22.08±4.32 ^a^ | 705.24±56.84 ^b^ |
|  | Females | 0.91±0.14 ^a^ | 17.00±1.95 ^a^ | 22.90±4.01 ^a^ | 1465.77±276.04* ^b^ |
| IL-1β | Males | 1.30±0.14 ^a^ | 2.10±0.15 ^b^ | 2.52±0.15 ^b^ | 2.07±0.21 ^b^ |
|  | Females | 0.76±0.05 ^a^ | 1.38±0.12 ^b^ | 1.57±0.12 ^b^ | 1.47±0.16 ^b^ |
| IL-6 | Males | 1.41±0.24 ^a^ | 41.98±3.75 ^b^ | 48.88±2.75 ^b^ | 37.00±2.68 ^b^ |
|  | Females | 1.39±0.30 ^a^ | 48.09±4.14 ^b^ | 60.01±5.18* ^b^ | 30.65±3.53 ^d^ |
| IL-10 | Males | 5.51±1.79 ^a^ | 2.27±0.56 ^a^ | 2.00±0.42 ^a^ | 16.49±1.97 ^b^ |
|  | Females | 1.56±0.40 ^a^ | 2.89±0.66 ^a^ | 4.16±1.03 ^a^ | 18.44±2.73 ^b^ |
| IL-12(p70) | Males | 2.20±0.49* ^a^ | 1.58±0.26 ^a^ | 1.00±0.18 ^a^ | 1.95±0.24 ^a^ |
|  | Females | 1.11±0.29 ^a^ | 1.41±0.33 ^a^ | 1.89±0.50 ^a^ | 2.42±0.49 ^a^ |
| TNF-α | Males | 1.64±0.14 ^a^ | 15.51±2.18 ^b^ | 20.58±1.71 ^b^ | 36.598±2.58 ^c^ |
|  | Females | 2.18±0.39 ^a^ | 28.00±4.07* ^b^ | 47.01±4.12* ^c^ | 68.31±5.88* ^d^ |
| TGF-β1 | Males | 1.25±0.15 ^a^ | 0.88±0.09 ^a^ | 1.21±0.09 ^a^ | 0.94±0.13 ^a^ |
|  | Females | 0.82±0.09 ^a^ | 0.72±0.11 ^a^ | 0.90±0.11 ^a^ | 1.14±0.16 ^a^ |

Data (mean ± SEM) are represented as the concentrations of proteins at Days 1-7p.i. relative to concentrations at Day 0, which was calculated because concentrations of some proteins (e.g., CCL3, TNF-α, and TGF-β) differed significantly between the sexes prior to infection and, therefore, reduced within-group variability in order to assess between-group differences. Data were analyzed with 2-way ANOVAs followed by Bonferroni t-tests with significant differences between the sexes at an individual time-point represented by an asterisk (*) and significant differences within a sex across time-points p.i. represented by different letters, *P* <0.05.
