# Supplementary material for: Elevated 17β-Estradiol Protects Females from Influenza A Virus Pathogenesis by Suppressing Inflammatory Responses
Source: PLoS Pathog. 2011 Jul 28;7(7):e1002149. doi: 10.1371/journal.ppat.1002149 (PMC3145801; doi:10.1371/journal.ppat.1002149)
Supplement: Table S2 — Fold induction of cytokines and chemokines in the lungs of males that were gonadally intact, gonadectomized, or gonadectomized with testosterone replaced. (DOC) [file ppat.1002149.s002.doc]

**Supporting Information**

**Table S2.** **Fold induction of cytokines and chemokines in lung homogenates from males that were gonadally intact (sham), gonadectomized (gdx), or gdx with testosterone (T) replaced.**

|  |  | **Days Post-Inoculation** | | | |
| --- | --- | --- | --- | --- | --- |
| **Cytokine** | **Treatment** | 1 | 3 | 5 | 7 |
| CCL2 | Sham | 10.61±9.55 ^a^ | 64.40±17.54 ^a^ | 73.70±18.07 ^b^ | 64.69±17.14 ^a^ |
|  | Gdx | 1.12±0.90 ^a^ | 20.06±7.32 ^a^ | 23.40±6.01 ^b^ | 30.46±4.19 ^b^ |
|  | Gdx + T | 0.83±0.19 ^a^ | 34.75±5.05 ^a, b^ | 72.62±23.28 ^b,c^ | 95.95±16.65 ^c,d^ |
| CCL3 | Sham | 0.84±0.14 ^a^ | 5.67±0.85 ^a^ | 11.27±2.81 ^a^ | 33.29±7.18 ^b^ |
|  | Gdx | 0.76±0.27 ^a^ | 3.08±0.55 ^a^ | 8.92±1.25 ^a^ | 41.52±4.32 ^b^ |
|  | Gdx + T | 1.13±0.23 ^a^ | 6.75±0.5 ^a^ | 23.19±8.07 ^b^ | 73.57±9.60* ^c^ |
| IFN-γ | Sham | 3.86±2.77 ^a^ | 24.19±6.55 ^a^ | 624.41±606.90 ^a^ | 3468.93±887.89 ^b^ |
|  | Gdx | 0.45±0.27 ^a^ | 9.23±2.19 ^a^ | 8.42±2.14 ^a^ | 828.26±157.16* ^a^ |
|  | Gdx + T | 1.06±0.2 ^a^ | 33.95±8.85 ^a^ | 29.75±7.39 ^a^ | 1773.98±589.72* ^b^ |
| IL-1β | Sham | 0.91±0.15 ^a^ | 1.62±0.19 ^b^ | 1.62±0.23 ^b^ | 1.16±0.11 ^a^ |
|  | Gdx | 1.00±0.12 ^a^ | 1.90±0.23 ^b^ | 2.21±0.12 ^b^ | 1.70±0.13 ^b^ |
|  | Gdx + T | 1.00±0.15 ^a^ | 1.82±0.22 ^a^ | 2.25±0.42 ^b^ | 1.50±0.11 ^a^ |
| IL-6 | Sham | 16.35±15.62 ^a^ | 198.60±36.53 ^a^ | 351.96±117.34 ^a^ | 213.97±111.77 ^a^ |
|  | Gdx | 0.77±0.70 ^a^ | 19.01±7.69 ^a^ | 29.87±8.09 ^b^ | 11.16±2.06 ^a^ |
|  | Gdx + T | 1.07±0.27 ^a^ | 123.35±24.96 ^a^ | 381.96±162.65 ^b^ | 146.72±34.64 ^a^ |
| IL-10 | Sham | 0.35±0.15 ^a^ | 0.23±0.09 ^a^ | 0.87±0.39 ^a^ | 9.77±3.62 ^a^ |
|  | Gdx | 0.70±0.04 ^a^ | 0.77±0.06 ^a^ | 0.73±0.08 ^a^ | 72.52±17.12* ^b^ |
|  | Gdx + T | 1.50±0.53 ^a^ | 2.12±1.06 ^a^ | 2.12±1.16 ^a^ | 57.43±15.73* ^b^ |
| IL-12(p70) | Sham | 0.83±0.23 ^a^ | 1.15±0.43 ^a^ | 1.64±0.43 ^a^ | 2.21±0.60 ^a^ |
|  | Gdx | 1.18±0.37 ^a^ | 1.47±0.50 ^a^ | 1.56±0.60 ^a^ | 1.69±0.47 ^a^ |
|  | Gdx + T | 1.29±0.23 ^a^ | 2.18±0.49 ^a^ | 2.05±0.53 ^a^ | 3.61±1.38 ^a^ |
| TGF-β1 | Sham | 0.81±0.12 ^a^ | 0.84±0.17 ^a^ | 1.17±0.26 ^a^ | 1.13±0.24 ^a^ |
|  | Gdx | 0.59±0.10 ^a^ | 0.53±0.05 ^a^ | 0.81±0.14 ^a^ | 0.70±0.13 ^a^ |
|  | Gdx + T | 0.95±0.15 ^a^ | 1.10±0.17 ^a^ | 0.85±0.15 ^a^ | 1.62±0.31 ^a^ |
| TNF-α | Sham | 1.46±0.67 ^a^ | 8.03±1.10 ^a^ | 13.26±1.94 ^b^ | 17.49±4.56 ^b^ |
|  | Gdx | 0.88±0.32 ^a^ | 5.51±0.39 ^b^ | 10.16±1.41 ^c^ | 14.31±1.17 ^d^ |
|  | Gdx + T | 1.11±0.23 ^a^ | 13.10±2.75 ^a,b^ | 19.60±4.59 ^b,c^ | 30.00±4.81 ^c,d^ |

Data are represented as the mean ± SEM. Data were analyzed with 2-way ANOVAs followed by Bonferroni t-tests, with significant differences compared with sham males at an individual time-point represented by an asterisk (*) and significant differences within a treatment group, across time-points p.i. represented by different letters, *P* <0.05.
