# Supplementary material for: Elevated 17β-Estradiol Protects Females from Influenza A Virus Pathogenesis by Suppressing Inflammatory Responses
Source: PLoS Pathog. 2011 Jul 28;7(7):e1002149. doi: 10.1371/journal.ppat.1002149 (PMC3145801; doi:10.1371/journal.ppat.1002149)
Supplement: Table S3 — Fold induction of cytokines and chemokines in the lungs of females that were gonadally intact, gonadectomized, or gonadectomized with estradiol replaced. (DOC) [file ppat.1002149.s003.doc]

**Supporting Information**

**Table S3.** **Fold induction of cytokines and chemokines in lung homogenates from females that were gonadally intact (sham), gonadectomized (gdx), or gdx with estradiol (E2) replaced.**

|  |  | **Days Post-Inoculation** | | | |
| --- | --- | --- | --- | --- | --- |
| **Cytokine** | **Treatment** | 1 | 3 | 5 | 7 |
| CCL2 | Sham | 0.84±0.27^a^ | 50.40±9.85 ^a^ | 119.09±28.20 ^b^ | 185.94±20.21 ^c^ |
|  | Gdx | 9.60±8.88 ^a^ | 54.51±24.56 ^a^ | 66.74±19.32 ^a^ | 148.49±26.58 ^b^ |
|  | Gdx + E2 | 0.03±0.02 ^a^ | 3.51±1.63 ^a^ | 11.42±6.65* ^a^ | 8.74±1.80* ^a^ |
| CCL3 | Sham | 0.81±0.09 ^a^ | 4.64±0.81 ^a^ | 10.24±1.01 ^a^ | 48.62±7.87 ^b^ |
|  | Gdx | 0.91±0.17 ^a^ | 3.77±0.58 ^a^ | 8.25±1.38 ^a^ | 33.45±3.45* ^b^ |
|  | Gdx + E2 | 1.41±0.50 ^a^ | 6.52±1.31 ^a^ | 14.72±2.17 ^a^ | 68.51±10.16* ^b^ |
| IFN-γ | Sham | 0.76±0.13 ^a^ | 12.52±2.88 ^a^ | 21.76±4.89 ^a^ | 3272.06±533.94 ^b^ |
|  | Gdx | 115.51±115.32 ^a^ | 4.45±0.94 ^a^ | 5.18±0.91 ^a^ | 390.27±139.65* ^a^ |
|  | Gdx + E2 | 0.00±0.0 ^a^ | 0.03±0.01 ^a^ | 0.07±0.02 ^a^ | 8.11±2.85* ^a^ |
| IL-1β | Sham | 0.94±0.21 ^a^ | 1.53±0.26 ^a^ | 1.76±0.27 ^a^ | 1.63±0.15 ^a^ |
|  | Gdx | 0.87±0.14 ^a^ | 1.41±0.14 ^a^ | 2.01±0.18 ^b^ | 1.42±0.20 ^a^ |
|  | Gdx + E2 | 0.74±0.10 ^a^ | 1.36±0.20 ^a^ | 1.75±0.29 ^b^ | 1.60±0.13 ^a^ |
| IL-6 | Sham | 1.36±0.24 ^a^ | 132.87±16.86 ^a^ | 590.59±199.94 ^a^ | 218.08±30.01 ^a^ |
|  | Gdx | 10.18±9.39 ^a^ | 137.51±39.79 ^a^ | 338.12±142.21 ^b^ | 127.61±25.06 ^a^ |
|  | Gdx + E2 | 0.02±0.01 ^a^ | 2.51±0.80 ^a,c^ | 7.92±1.89 ^b^ | 6.56±1.14 ^b,c^ |
| IL-10 | Sham | 1.16±0.15 ^a^ | 1.38±0.32 ^a^ | 2.65±0.68 ^b^ | 93.45±21.77 ^c^ |
|  | Gdx | 2.59±2.35 ^a,b^ | 0.13±0.02 ^a^ | 0.48±0.15 ^a^ | 10.64±3.57 ^b^ |
|  | Gdx + E2 | 0.03±0.00 ^a^ | 0.06±0.02 ^a^ | 0.18±0.07 ^a^ | 3.98±1.23* ^a^ |
| IL-12(p70) | Sham | 1.68±0.44 ^a^ | 1.14±0.29 ^a^ | 2.45±0.65 ^a^ | 3.44±0.82* ^a^ |
|  | Gdx | 1.10±0.37 ^a^ | 1.00±0.29 ^a^ | 1.06±0.28 ^a^ | 1.84±0.55 ^a^ |
|  | Gdx + E2 | 0.67±0.17 ^a^ | 0.72±0.16 ^a^ | 0.88±0.28 ^a^ | 1.44±0.33 ^a^ |
| TNF-α | Sham | 1.10±0.18 ^a^ | 11.74±1.33 ^b^ | 21.80±2.86 ^c^ | 36.77±2.88 ^d^ |
|  | Gdx | 3.97±3.03 ^a^ | 8.21±0.94 ^a^ | 15.88±2.46 ^b^ | 24.53±1.47 ^c^ |
|  | Gdx + E2 | 0.16±0.01 ^a^ | 0.95±0.23* ^a^ | 2.64±0.54* ^a^ | 3.54±0.93* ^a^ |
| TGF-β1 | Sham | 0.99±0.22 ^a^ | 0.88±0.10 ^b^ | 1.61±0.11 ^c^ | 1.40±0.15 ^d^ |
|  | Gdx | 0.97±0.16 ^a^ | 0.99±0.16 ^a^ | 1.01±0.17 ^b^ | 1.13±0.18 ^c^ |
|  | Gdx + E2 | 1.33±0.11 ^a^ | 1.65±0.23 ^a^ | 1.37±0.14 ^a^ | 1.99±0.33 ^a^ |

Data are represented as the mean ± SEM. Data were analyzed with 2-way ANOVAs followed by Bonferroni t-tests, with significant differences compared with sham females at an individual time-point represented by an asterisk (*) and significant differences within a treatment group, across time-points p.i. represented by different letters, *P* <0.05.
