# Supplementary material for: Elevated 17β-Estradiol Protects Females from Influenza A Virus Pathogenesis by Suppressing Inflammatory Responses
Source: PLoS Pathog. 2011 Jul 28;7(7):e1002149. doi: 10.1371/journal.ppat.1002149 (PMC3145801; doi:10.1371/journal.ppat.1002149)
Supplement: Table S4 — Fold induction of cytokines and chemokines in the lungs of females that were gonadectomized and assigned to receive vehicle, estradiol, an ERα agonist, or an ERβ agonist. (DOC) [file ppat.1002149.s004.doc]

**Supporting Information**

**Table S4.** **Fold induction of cytokines and chemokines in lung homogenates from females that were gonadectomized (gdx) and assigned to receive vehicle, estradiol (E2), an ERα agonist (PPT), or an ERβ agonist (DPN).**

|  | **Treatment** | | | |
| --- | --- | --- | --- | --- |
| **Cytokine** | Gdx+Vehicle | Gdx + E2 | Gdx + ERα Agonist | Gdx + ERβ Agonist |
| IFN-γ | 3738.71±880.88 | 2057.50±86.99 | 1859.03±199.42 | 2356.71±281.15 |
| IL-6 | 979.14±134.86 | 864.76±139.70 | 390.28±72.70*† | 674.62±120.69 |
| IL-10 | 189.73±20.72 | 311.54±26.77* | 206.74±24.40† | 90.34±11.37*† |
| IL12(p70) | 4.29±.097 | 4.82±.43 | 4.55±.052 | 5.43±.57 |

Data are represented as the mean ± SEM. Data were analyzed with 1-way ANOVAs followed by Bonferroni t-tests with significant differences compared with vehicle-treated females represented by an asterisk (*) and significant differences compared with E2-treated females represented by a dagger (†) *P* <0.05.
